# Divergences in antihypertensive therapy in special situations in nephrology

**DOI:** 10.1590/S1516-31802008000100007

**Published:** 2008-01-03

**Authors:** Marcelo Montebello Lemos, Alessandra Coelho Pedrosa, Alze Pereira Tavares, Miguel Ângelo Góes, Sérgio Antônio Draibe, Ricardo Sesso

**Keywords:** Hypertension, Antihypertensive agents, Kidney diseases, Guidelines, Evidence-based medicine, Hipertensão, Anti-hipertensivos, Nefropatias, Diretrizes, Medicina baseada em evidências

## Abstract

**CONTEXT AND OBJECTIVE:**

The choice of an antihypertensive drug is based on several criteria and specific situations give rise to doubt and controversy. The aim here was to evaluate physicians’ approaches towards treatment with antihypertensive agents in specific situations.

**DESIGN AND SETTING:**

Cross-sectional study, at Universidade Federal de São Paulo, São Paulo.

**METHODS:**

A questionnaire was applied during a nephrology meeting to evaluate individual approaches towards each hypothetical clinical situation. The questionnaire consisted of five multiple-choice questions (clinical cases) concerning controversial aspects of antihypertensive therapy.

**RESULTS:**

A total of 165 questionnaires were analyzed. Most participants were nephrologists (93.2%). There was a preference for angiotensin-converting enzyme (ACE) inhibitors in at least two of the cases. Only 57.2% of the physicians were correct in choosing beta-blockers as the first-line drugs for patients with ischemic coronary disease. Moreover, 66.2% chose ACE inhibitors as the first-line drugs for patients with chronic kidney disease and proteinuria. About 5% of the physicians did not follow the current recommendations for the use of ACE inhibitors in diabetic patients with microalbuminuria. The most controversial question concerned the first-line drug for advanced chronic kidney disease. Most physicians were correct in choosing calcium channel blockers and avoiding ACE inhibitors in renovascular hypertension in the case of a patient with a single functioning kidney.

**CONCLUSIONS:**

Most physicians adopted the correct approach, but some had an alternative strategy for the same situations that was not based on evidence.

## RESUMO

**CONTEXTO E OBJETIVO:**

A escolha da droga anti-hipertensiva depende de diversos fatores. Determinadas situações geram dúvida e discordância entre médicos. O objetivo foi avaliar a conduta de nefrologistas e clínicos em situações hipotéticas relacionadas ao tratamento da hipertensão.

**TIPO DE ESTUDO E LOCAL:**

Estudo transversal, na Universidade Federal de São Paulo, São Paulo, Brasil.

**MÉTODO:**

Foi aplicado um questionário com cinco casos clínicos hipotéticos durante o X Encontro Paulista de Nefrologia, com o objetivo de avaliar a primeira escolha de droga anti-hipertensiva em cada situação, contemplando as principais dúvidas relacionadas.

**RESULTADOS:**

Foram analisados 165 questionários. A maior parte dos médicos era composta por nefrologistas (93,2%). Houve preferência pelo uso dos inibidores da enzima conversora de angiotensina (IECA) em dois dos cinco casos. Apenas 57,2% acertaram na escolha do beta-bloqueador como primeira opção nos pacientes com coronariopatia. Além disso, 66,2% optaram por IECA como drogas de eleição em renais crônicos com proteinúria. Aproximadamente 5% dos colegas não seguiram as recomendações quanto ao uso dos IECA ou ARA em diabéticos com microalbuminúria. A questão com mais divergências foi a que avaliou a droga de escolha na doença renal crônica em fase avançada. A maior parte dos médicos acertou ao evitar o uso dos IECA na hipertensão renovascular em pacientes com rim único funcionante.

**CONCLUSÕES:**

A maior parte dos colegas adota condutas coerentes com os consensos relacionados à hipertensão arterial e doença renal, contudo, uma parcela não desprezível diverge e adota condutas não recomendadas.

## INTRODUCTION

Treatment of arterial hypertension represents one of the main strategies for preventing target organ injuries in patients with hypertension, such as in cases of chronic kidney disease (CKD) and heart disease.^1,2^ In parallel to progressive reductions in target blood pressure as a preventive measure, doubts remain regarding the most adequate drug classes for each type of patient or clinical situation.^1,3^ Divergences in the approaches towards treating hypertension exist among health professionals from different fields and even among those within the same specialty, but these differences in clinical practice have not been quantified. In addition, in certain situations, the data in the literature do not reflect unanimity. Some of these situations were the focus of the present study, in which the objective was to evaluate the approaches adopted by physicians, and particularly nephrologists, towards treatments involving the use of antihypertensive drugs in controversial situations and to discuss the evidence in the literature regarding these topics.

## OBJECTIVE

The aim was to evaluate physicians’ approaches towards treatment using antihypertensive agents in specific situations. In addition, the results were discussed based on the current scientific evidence.

## METHODS

A questionnaire (Appendix 1) consisting of five hypothetical clinical cases of patients in different clinical situations, with a formal or relative indication for the use of antihypertensive drugs, was applied during a nephrology meeting (Tenth São Paulo Nephrology Meeting) that was held from September 14 to 17, 2005. The questionnaire was applied to nephrologists and other clinicians participating in the meeting who agreed to respond to the questions. There was no time limit for completing the questionnaire, and the participants were asked to choose only one response from among the alternatives offered. Questions with multiple answers were excluded from the analysis.

The cases presented comprised topics of interest to physicians who care for patients with hypertension and/or kidney disease, and the participants were asked what their first choice of antihypertensive drug would be in the following situations: patients with coronary disease without renal injury (question 1), nondiabetic patients with CKD and significant proteinuria (question 2), diabetic patients with microalbuminuria (question 3), patients with advanced CKD (question 4) and patients with renovascular hypertension and a single functioning kidney (question 5).

Some characteristics of the qualifications of the physicians who answered the questionnaire were also evaluated, including medical specialty, presence or absence of specialization or medical residency, specialist title, and year of graduation.

Descriptive statistical analysis was performed using the SPSS 11.0 software. Values were expressed as absolute numbers and percentages of valid responses. The year of graduation was reported as the median and range. The topic or central element of each case has been discussed based on the evidence in the literature.

## RESULTS

A total of 165 physicians answered to the questionnaire. Most of them were nephrologists (93.2%), followed by general clinicians (3.1%) and physicians of other clinical specialties (3.7%). The median year of graduation was 1990 (range: 1953-2003). Most of the physicians had a specialist title or had completed medical residency (81.6%), and 11.4% were currently enrolled in a medical residency program.

### Question 1

The answers to question 1, which evaluated the hypothetical situation of a 42-year-old hypertensive woman with coronary disease, normal renal function and negative microalbuminuria, are summarized in Table 1.

**Table 1.** Antihypertensive drugs chosen by physicians in five different nephrological clinical situations

Drug	Question 1	Question 2	Question 3	Question 4	Question 5
	n (%)				
Beta-blockers	**91 (57.2)**	2 (1.3)	-	7 (4.6)	7 (4.5)
ACE inhibitors	28 (17.6)	**102 (66.2)**	**128 (84.8)**	37 (24.4)	38 (24.6)
Diuretics	17 (10.7)	5 (3.2)	2 (1.3)	28 (18.4)	11 (7.1)
Calcium channel blockers	15 (9.4)	24 (15.6)	-	**64 (42.1)**	**78 (50.3)**
ARA	6 (3.8)	17 (11.0)	17 (11.2)	9 (5.9)	12 (7.7)
Peripheral vasodilators	2 (1.3)	1 (0.7)	-	3 (2.0)	5 (3.2)
Central sympatholytic agents	-	2 (1.3)	1 (0.7)	4 (2.6)	4 (2.6)
None	-	-	3 (2.0)	-	-
Others	-	1 (0.7)	-	-	-
**Total**	**159 (100)**	**154 (100)**	**151 (100)**	**152 (100)**	**155 (100)**

ACE = angiotensin-converting enzyme; ARA = angiotensin receptor antagonists.

The most frequent answers to each question are highlighted in bold.

Most (57.2%) of the 159 physicians who gave valid answers opted for beta-blockers as the first-line drugs in this situation, followed by 17.6% who chose angiotensin-converting enzyme (ACE) inhibitors, 10.7% who indicated diuretics as their first choice and 9.4% who chose calcium channel blockers. Six answers were not valid and were not included in the statistical analysis.

### Question 2

Question 2 evaluated the situation of a 40-year-old hypertensive and nondiabetic man with CKD (creatinine: 3.0 mg/dl; estimated clearance: 30 ml/min; and proteinuria: 1.5 g/24 h). The answers are summarized in Table 1.

Among the 154 nephrologists who answered this question, 66.2% chose ACE inhibitors as the first-line drugs, 15.6% calcium channel blockers and 11% angiotensin receptor antagonists (ARA).

### Question 3

The hypothetical situation of a 50-year-old normotensive man with type I diabetes, normal renal function and microalbuminuria (120 mg/24 h) was evaluated and the answers are shown in Table 1.

A total of 151 physicians answered this question; 128 (84.8%) of them indicated ACE inhibitors, 17 (11.2%) indicated ARA and varying proportions from 0.7 to 1.3% chose other hypotensive medications, while three (2.0%) of the participants responded that they would not use any antihypertensive medication in this situation.

### Question 4

The answers to question 4, which evaluated the situation of a 60-year-old hypertensive nondiabetic man with CKD (creatinine: 4.2 mg/dl; estimated clearance: 14 ml/min; and proteinuria: 1.8 g/24 h), are shown in Table 1.

A total of 152 physicians answered this question and most opted for calcium channel blockers (42.1%), followed by ACE inhibitors and diuretics. Other alternatives were chosen at lower proportions, ranging from 2.0 to 5.9% of the valid answers.

### Question 5

Question 5 evaluated the situation of a 58-year-old hypertensive nondiabetic man with CKD (creatinine: 1.7 mg/dl; estimated clearance: 55 ml/min; and proteinuria: 1.4 g/24 h). Angiography by means of nuclear magnetic resonance demonstrated 75% left renal artery stenosis. The left kidney length was 10.8 cm and the right kidney length was 6.0 cm. The answers are summarized in Table 1.

A total of 155 physicians answered this question. Seventy-eight (50.3%) indicated calcium channel blockers as the first-line drug, while 38 (24.6%) indicated ACE inhibitors and varying proportions from 2.6 to 7.7% chose other hypotensive medications.

## DISCUSSION

### Essential hypertension, normal renal function and ischemic coronary disease

In patients with coronary disease, anti-hypertensive treatment should be aimed at reducing symptoms, increasing exercise tolerance and reducing morbidity and mortality. Beta-blockers are recommended by the American Heart Association/American College of Cardiology as the first-line drugs in these cases because they reduce cardiac work (due to their chronotropic and negative inotropic effects and a reduction in afterload).^4^ These drugs reduce mortality in some groups, including patients with a history of previous infarction, patients undergoing revascularization surgery or angioplasty and patients with ischemic coronary disease and left ventricular dysfunction, as demonstrated in the metoprolol in dilated cardiomyopathy (MDC) trial and in the metoprolol controlled release/extended release randomized intervention trial in chronic heart failure (MERIT-HF) studies, among others.^5–9^

Based on the information given in this question, the main doubt relates to the patient's cardiac function since, if left ventricular dysfunction of ischemic etiology were characterized, there would be two correct answers. In this situation, beta-blockers are still indicated, in addition to the use of ACE inhibitors, in order to prevent myocardial remodeling and to help in controlling the postload, thereby improving cardiac performance and decreasing mortality. ACE inhibitors exert an additive effect on the beta-blocker in this case and neither drug alone is more efficient than the combination of both.^10^ The benefit of ACE inhibitors in cases of stable angina with preserved systolic function and without left ventricular hypertrophy is debatable and conflicting results have been reported in the literature. Thus, ACE inhibitors remain as second-line drugs.^11,12^

Calcium channel blockers are effective in cases of stable angina, but dihydropyridines may cause reflex tachycardia, with consequent worsening or precipitation of symptoms. Dihydropyridines should be avoided in patients with previous infarction and non-dihydropyridines are generally used as an alternative to beta-blockers in patients with intolerance or contraindication against them. Non-dihydropyridines may also be used in combination with beta-blockers in patients in whom anginal symptoms persist despite beta-blockade.^13,14^

### Nondiabetic patients with chronic kidney disease and proteinuria higher than 1 g/24 h

Antihypertensive therapy is fundamental for renal protection and the introduction of drugs that directly act on the renin-angiotensin-aldosterone (RAA) system has led to advances in the field of nephroprotection. These drugs not only exert an antihypertensive effect but also perform antiproteinuric and growth factor-modulating activities. Proteinuria is an independent risk factor for the deterioration of renal function.^15,16^ The modification of diet in renal disease (MDRD) study demonstrated that presence of proteinuria was a determining factor for the renoprotective effect found with reduced blood pressure, since the additional benefit of lower pressure levels was more pronounced in patients with proteinuria.^17^ The level of reduction in proteinuria, as well as the residual proteinuria over the course of antihypertensive treatment, is also of prognostic value. In nondiabetic patients, Aperloo et al. demonstrated that the antiproteinuric response to treatment with enalapril was predictive of the decline in renal function and that the presence of residual proteinuria during treatment with ACE inhibitors or beta-blockers was associated with subsequent worsening of renal function.^18^

Large randomized trials were subsequently conducted and confirmed these data. The ramipril efficacy in nephropathy (REIN) study demonstrated that ramipril was superior to placebo in reducing the decline of renal function and proteinuria in both the group with initial proteinuria higher than 3 g/24 h and in the group with proteinuria ranging from 1 to 3 g/24 h.^19^ Also in nondiabetic patients, the angiotensin-converting-enzyme inhibition on progressive renal insufficiency (AIPRI) study confirmed the superiority of angiotensin-converting-enzyme (ACE) inhibitors over conventional antihypertensive drugs in attenuating the loss of renal function.^20^ Recently, the COOPERATE study, comparing the combination of trandolapril and losartan with the use of either drug alone among 230 nondiabetic patients, demonstrated that the risk of renal function deterioration was reduced by about 50% with the combination of the two drugs, compared with monotherapy with either drug alone.^21^

In conclusion, ACE inhibitors and ARA are the classes with greatest nephroprotective and antiproteinuric benefits, and both of these are first-line drugs in the situation described.

### Type 1 diabetes mellitus, with microalbuminuria and normal blood pressure

Microalbuminuria, in addition to being a marker for early diabetic nephropathy, is also associated with a higher cardiovascular risk.^22,23^ In type 1 diabetes mellitus, progression from microalbuminuria to nephropathy with proteinuria (> 300 mg/24 h) generally occurs 10-15 years after the diagnosis.^24^

In addition to good glycemic control, rigorous blood pressure control, preferentially with the use of an ACE inhibitor, is necessary in order to control microalbuminuria and slow down the progression to proteinuria. This class of drug is even indicated in normotensive diabetic patients with microalbuminuria since their beneficial effect does not depend on the presence of systemic arterial hypertension but is due to their local and intraglomerular hemodynamic effects and to their ability to restore glomerular permselectivity.^25–27^

Studies comparing captopril and placebo in diabetic patients with microalbuminuria reported a lower risk of progression to proteinuria in the captopril group.^25,26^ These results were reproduced with perindopril, in comparison with nifedipine or placebo.^28^ A prospective study evaluating factors associated with a reduction in microalbuminuria (and not with progression to proteinuria) identified good glycemic control and control over blood pressure levels and the lipid profile as determining factors. However, the use of ACE inhibitors was not associated with a reduction in microalbuminuria.^29^

The combination of an ACE inhibitor with an ARA has been shown to produce a greater reduction in proteinuria and cardiovascular risk than does monotherapy (ACE inhibitor or ARA), thus demonstrating that the nephroprotective effects of these drugs are additive.^30,31^ Nevertheless, regarding microalbuminuria, there is no evidence indicating that the effect of the combination of these drugs is superior to that of each individual drug. Most participants who responded to question 3 provided an adequate answer. Surprisingly, 2.0% of the physicians did not indicate any pharmacological treatment and 2.0% opted for other drugs as the first-line treatment.

### Advanced chronic kidney disease

One frequent doubt relates to whether or not there is any renal function limit in relation to the use of drugs that directly act on the RAA system, due to the risk of deterioration of renal function. The Benazepril study demonstrated that ACE inhibitors had a beneficial effect on the progression of CKD.^20^ The REIN study confirmed these results, by firstly demonstrating a reduction in the progression rate of CKD in patients with significant proteinuria (> 3 g/24 h) who were taking ramipril (an ACE inhibitor). In addition, a secondary analysis showed that this beneficial effect extended to patients with proteinuria < 3 g/24 h.^19^ The most interesting result from that study was the demonstration that even patients in the lower third percentile of renal function (glomerular filtration rate of 11 to 33 ml/min) benefited from the use of ACE inhibitors. However, the heterogeneity of the group with poor renal function, which included patients in CKD stages III, IV and V, should be emphasized and it should be remembered that the expected decline in renal function (known as reset) after blockade of the RAA system has different impacts on different CKD stages.

The RENAAL study evaluated type 2 diabetic patients with chronic nephropathy and reported worsening of renal outcomes (progression to dialytic CKD) in patients with the poorest renal function, i.e. individuals with a creatinine concentration of 3.6 mg/dl or more.^32,33^ One of the largest clinical trials investigating the treatment of arterial hypertension, the antihypertensive and lipid-lowering treatment to prevent heart attack trial (ALLHAT study), did not find any difference in nephroprotection between groups using chlorthalidone, lisinopril and amlodipine.^34^ However, the ALLHAT study did not focus on advanced CKD.

The consensus of the Brazilian Society of Hypertension recommends ACE inhibitors for patients with creatinine less than 3 mg/dl, and particularly for those with proteinuria and/or diabetes, but suggests caution if prescribing these drugs to patients with creatinine higher than 3 mg/dl.^3^ In the latter case, creatinine should be reevaluated within a period of one week. We emphasize that the suggestion of a cutoff value for creatinine without taking into account creatinine clearance may lead to erroneous evaluation of renal function in some patients.

The Joint National Committee on Prevention, Detection, Evaluation and Treatment of High Blood Pressure also has not defined a renal function limit in relation to the use of ACE inhibitors, but has suggested that an increase of up to 35% in creatinine, compared with baseline levels, might be tolerated and would not justify discontinuation of the drug.^1^ The dialysis outcomes quality initiative (DOQI) guidelines indicate ACE inhibitors and ARA as the first-line drugs for patients with diabetic nephropathy as the etiology of kidney disease, and for all other etiologies when proteinuria is present.^2^ However, the guidelines do not mention the renal function level as a limiting factor in introducing these drug classes and only recommend caution at the beginning of treatment, with monitoring of renal function and potassemia.

The concern regarding potential increases in creatinine following the introduction of ACE inhibitors contrasts with the beneficial effects of this class of antihypertensive drugs and explains the heterogeneity in the choice of antihypertensive agent in cases of patients with advanced CKD.^35^ Hou et al. suggested that the administration of ACE inhibitors to patients with creatinine levels ranging from 3.0 to 5.0 mg/dl is safe and beneficial with regard to nephroprotection.^36^ An editorial referring to this same article suggested that ACE inhibitors should be administered in a single morning dose in the case of patients with a tendency toward hyperkalemia, since this strategy favors potassium excretion during the night.^37^

In conclusion, ACE inhibitors are the first-line treatment for patients with CKD and there is no consensus regarding a renal function limit for the use of these drugs. However, advanced CKD places the patient at a high risk of additional decline in renal function and precipitation of uremic symptoms, hyperkalemia or the need for dialysis. We believe that, at this stage of kidney disease, nephroprotection loses its importance but the cardiovascular benefit should still be taken into account. Thus, if medication is indicated, drug treatment should be started at a low dose (25% of the full dose) and renal function and potassemia should be monitored.

### Unilateral renal artery stenosis in patients with a single functioning kidney

Patients with renal artery stenosis mainly comprise individuals with essential arterial hypertension and/or atherosclerotic disease. Antihypertensive therapy, reduction of LDL cholesterol and cessation of smoking are the main measures for treating these patients, in addition to renal revascularization.^38^ The Seventh Report of the Joint National Committee on Prevention, Detection, Evaluation and Treatment of High Blood Pressure recommended that patients with abnormal renal function should keep their blood pressure levels lower than 130 x 80 mmHg, whereas K/DOQI established that blood pressure levels should be lower than 125 x 75 mmHg for patients with proteinuria above 1 g/24 h.^1,2^

Many patients with renal artery disease present reduced renal function and therefore selection of the antihypertensive drug should take into account not only the presence of renal artery stenosis but also whether it is unilateral or bilateral, as well as possible aggravating conditions such as proteinuria, left ventricular hypertrophy and heart failure. Thus, many patients with renovascular disease will qualify for treatment with ACE inhibitors and/or ARA, because the survival of patients with renovascular hypertension has been shown to be better when an ACE inhibitor is part of the treatment.^38,39^

The major concern regarding the use of ACE inhibitors for renovascular hypertension is their potential for causing acute renal failure.^40^ The mechanism is related to the inhibition of angiotensin II-mediated compensatory mechanisms that develop from the stenotic lesion. In addition, a decreased glomerular filtration rate (GFR) at the beginning of antihypertensive treatment is not an effect caused exclusively by ACE inhibitors. Any medication that reduces blood pressure sufficiently such that it reduces the blood flow beyond the stenotic lesion, thereby overcoming the capacity for renal autoregulation, may cause a decline in renal function.^41^ Caution at the onset of treatment with ACE inhibitors is important for patients with suspected or known renal artery stenosis, with the need for monitoring renal function and serum potassium levels.^42^ A significant reduction in GFR (more than 30%, or an increase of > 0.5 mg/dl in serum creatinine) during treatment with ACE inhibitors is observed in a minority of patients, generally those with bilateral renal artery stenosis or renal artery stenosis in a solitary kidney.^2,38,43^ In situations in which the decrease in GFR exceeds these limits, the drug should be discontinued and renal revascularization might be required.^38^ If monotherapy with an ACE inhibitor or ARA is poorly tolerated or adequate pressure control is not achieved, other antihypertensive agents could be administered in combination, such as calcium channel blockers that preferentially dilate the afferent arteriole and do not alter GFR.^2^

The fundamental objective in treatment for renovascular hypertension is to preserve renal function and blood pressure control.^44^ In the present hypothetical situation (case 5), the use of ACE inhibitors may trigger acute renal failure. Another safer option before renal revascularization is the use of a calcium channel blocker.

## CONCLUSIONS

Differences in the approaches towards treatment using antihypertensive drugs exist even among nephrologists. The lack of use of ACE inhibitors in situations with a classical indication such as diabetic patients with microalbuminuria, as well as their use in contraindicated situations such as significant renal artery stenosis in patients with a single functioning kidney, and their improper use in more advanced phases of kidney disease are worrisome and indicate the need for more detailed discussion of these topics and more widespread distribution of updated guidelines. Continuous medical training based on the best evidence available is fundamental and will tend to considerably reduce the morbidity and mortality relating to diseases such as hypertension and CKD.

## Appendix 1. Model of the questionnaire applied

**What is the drug of choice in the situations reported below?**

1. Woman, 42 years old, hypertensive, coronary disease, normal renal function, negative microalbuminuria.
2. Man, 40 years old, hypertensive, nondiabetic, chronic kidney disease (creatinine: 3.0 mg/dl; estimated clearance: 30 ml/min; and proteinuria: 1.5 g/24 h).
3. Man, 50 years old, normotensive, type I diabetes, normal renal function, microalbuminuria: 120 mg/24 h.
4. Man, 60 years old, hypertensive, nondiabetic, chronic kidney disease (creatinine: 4.2 mg/dl; estimated clearance: 14 ml/min; and proteinuria 1.8 g/24 h).
5. Man, 58 years old, hypertensive, nondiabetic, chronic kidney disease (creatinine: 1.7 mg/dl; estimated clearance: 55 ml/min; and proteinuria: 1.4 g/24 h). Angiography by nuclear magnetic resonance showing 75% left renal artery stenosis. Left kidney: 10.8 cm; right kidney: 6.0 cm.

**The alternatives for all cases are:**

Diuretics ( )
Beta-blockers ( )
Angiotensin-converting enzyme inhibitors ( )
Angiotensin antagonists ( )
Peripheral vasodilators ( )
Central sympatholytic agents ( )
Calcium channel blockers ( )
Others:____________________________

Specialty: Nephrology ( )
Others (specify):____________________
Do you have a residency or specialization title?
Yes ( )
No ( )
Ongoing ( )
Year of graduation:__________________
